# Overexpression of heparanase enhances T lymphocyte activities and intensifies the inflammatory response in a model of murine rheumatoid arthritis

**DOI:** 10.1038/srep46229

**Published:** 2017-04-12

**Authors:** Andreas Digre, Kailash Singh, Magnus Åbrink, Rogier M. Reijmers, Stellan Sandler, Israel Vlodavsky, Jin-Ping Li

**Affiliations:** 1 Department of Medical Biochemistry and Microbiology, University of Uppsala, The Biomedical Center, Box 582, SE-751 23 Uppsala, Sweden; 2 Department of Medical Cell Biology, Uppsala University, The Biomedical Center, Box 571, SE-751 23 Uppsala, Sweden; 3 Department of Biomedical Sciences and Veterinary Public Health, Section of Immunology, Swedish University of Agricultural Sciences, VHC, Box 7028, SE-75007 Uppsala, Sweden; 4 Department of Molecular Cell Biology and Immunology, VU University Medical Center, Amsterdam, The Netherlands; 5 Cancer and Vascular Biology Research Center, The Bruce Rappaport Faculty of Medicine, Technion, Haifa, Israel

## Abstract

Heparanase is an endo-glucuronidase that degrades heparan sulfate chains. The enzyme is expressed at a low level in normal organs; however, elevated expression of heparanase has been detected in several inflammatory conditions, e.g. in the synovial joints of rheumatoid arthritis (RA) patients. Herein, we have applied the model of collagen-induced arthritis (CIA) to transgenic mice overexpressing human heparanase (Hpa-tg) along with wildtype (WT) mice. About 50% of the induced animals developed clinical symptoms, *i.e.* swelling of joints, and there were no differences between the Hpa-tg and WT mice in the incidence of disease. However, Hpa-tg mice displayed an earlier response and developed more severe symptoms. Examination of cells from thymus, spleen and lymph nodes revealed increased innate and adaptive immune responses of the Hpa-tg mice, reflected by increased proportions of macrophages, antigen presenting cells and plasmacytoid dendritic cells as well as Helios-positive CD4^+^ and CD8^+^ T cells. Furthermore, splenic lymphocytes from Hpa-tg mice showed higher proliferation activity. Our results suggest that elevated expression of heparanase augmented both the innate and adaptive immune system and propagated inflammatory reactions in the murine RA model.

Rheumatoid arthritis (RA) is a chronic inflammatory autoimmune disease affecting articular and extra-articular tissues. The typical pathological changes in the synovia are marked infiltration of innate and adaptive immune cells, followed by aggressive proliferation of synovial tissue (ST), leading to destruction of bone and cartilage^1^. Entry of leukocytes into inflamed tissues is highly ordered and involves a series of adhesion receptors and ligands including heparan sulfate proteoglycans (HSPGs)^2^. HSPGs are glycoconjugates ubiquitously expressed on the cell surface or as extracellular matrix constituents, exerting diverse biological functions. HSPGs are composed of a core protein to which several heparan sulfate (HS) side chains are covalently attached. The HS side chains interact with a multitude of proteins including cytokines and chemokines^3^,^4^,^5^. The different biological functions of HS are linked to their molecular structures that are expressed in a spatial and temporal fashion. Therefore, an alteration in HS structure can affect its biological functions, as demonstrated in several inflammatory mouse models^6^,^7^,^8^.

Heparanase is an endo-glucuronidase that specifically cleaves HS, thereby modifying its molecular structures. This enzyme is expressed at low levels under healthy conditions, and is often upregulated in pathological situations, *e.g.* inflammation. Elevated expression of heparanase was found to be associated with several inflammatory conditions, such as pulmonary sepsis^9^, lung allergic inflammation^10^ and inflammatory bowel disease^11^. A dramatic increase in heparanase level (~100-fold) was detected in the synovial fluid and tissues from patients with RA, but not in osteoarthritis patients^12^. However, the pathophysiological role of elevated expression of heparanase in the joints of patients is unknown. Nonetheless, HSPGs have been found in chronically inflamed synovium^13^, and the chemokine CXCL12 is expressed at high levels in synovial tissues of RA and is displayed on endothelial cells along with HSPGs^14^. Taken together, current knowledge strongly suggests a role for HS and heparanase in the pathogenesis of RA.

In this study, we used transgenic mice overexpressing human heparanase (Hpa-tg)^15^ to examine the functional role of heparanase in a murine model for RA. By applying the collagen induced arthritis (CIA) model, we found that Hpa-tg mice displayed earlier and more severe clinical symptoms than WT mice. Assessment of cells from immune organs revealed higher proportions of innate and adaptive immune cells that have been shown to play pivotal roles during the early developmental stage of RA^16^,^17^. Taken together, our results suggest that heparanase may trigger and enhance both innate and adaptive immunity in response to inflammatory stimuli.

## Results

### Higher inflammatory reactions in mice overexpressing heparanase

**Figure 1:**
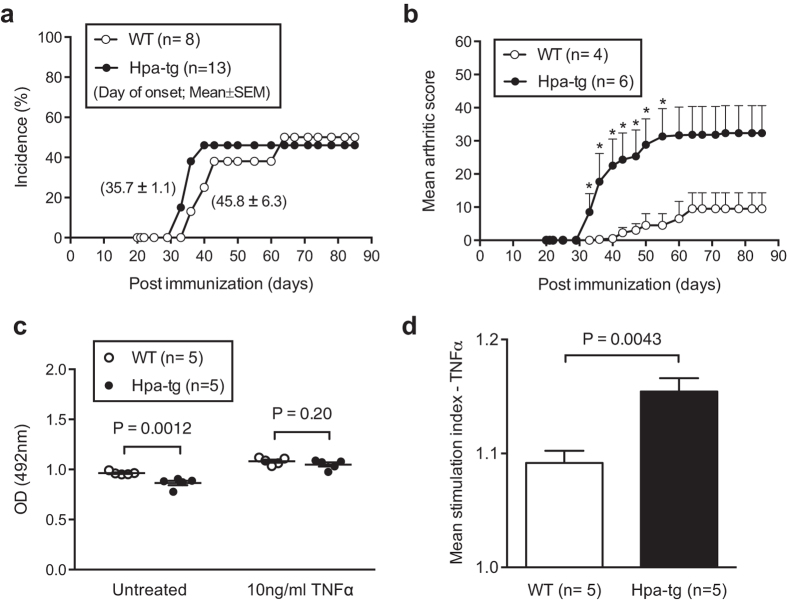
Severe inflammatory symptoms in Hpa-tg mice. (**a**) The incidence of paws swelling symptoms post primary and booster immunizations. (**b**) Mean arthritic score of paw swelling in arthritic mice only, with a maximum score of 60. The mean scores were significantly higher in Hpa-tg mice at the time points indicated by asterisks; *p < 0.05. The scores of each mouse are presented in Supplementary Table S1. (**c**) Proliferation of synovial fibroblasts (SFs) stimulated with 10 ng/ml TNFα was measured using the MTS assay. The data are mean value of triplicates from each mouse (n = 5). (**d**) Mean stimulation index of TNFα induced proliferation of SFs from WT and Hpa-tg mice. Error bars represent SEM.

To assess the effect of heparanase expression on the pathology of RA, we applied CIA to wild type (WT) mice and mice overexpressing human heparanase (Hpa-tg). Starting on week 3 after immunization, the mice were monitored daily for signs of arthritis through clinical scoring by visual inspection of the forelimbs and the hind paw swelling as suggested^18^. As expected, about 50% of WT mice developed symptoms and there was no difference in the incidence of arthritis development between the WT and Hpa-tg groups (Fig. 1a). The overall fairly low incidence rate reflects the genetic property of C57Bl/6 mice. However, in Hpa-tg mice symptoms appeared a few days earlier than in WT mice, with markedly higher scores (Fig. 1b and Supplementary Table S1). The pronounced inflammation in the joints of Hpa-tg mice was further evidenced by histological analysis of sections from the joints (Supplementary Fig. S1a), showing that infiltration of inflammatory cells and the tissue damage were correlated with the clinical score. Grading of the pathological parameters (bone erosion and cell infiltration) demonstrated an agreement between the clinical and pathological scores (Supplementary Fig. S1b). As control, no synovial infiltration of cells was observed in naïve Hpa-tg and WT mice, indicating that overexpression of heparanase, per se, did not induce autoimmune reactions or immune cell infiltration in these mice.

As synovial fibroblasts (SF) play important roles in the pathology of RA, we also examined the activity of SF isolated from naïve Hpa-tg and WT mice. Western blot analysis confirmed overexpression of heparanase in the cells isolated from Hpa-tg mice (Supplementary Fig. S2). Proliferation of non-stimulated SF from Hpa-tg mice was slower than that of SF from WT mice (Fig. 1c); however, the Hpa-tg SF showed increased proliferation upon TNF-alpha stimulation, exhibiting a significantly higher ‘stimulation index’ (Fig. 1d). As TNF-alpha is a major cytokine involved in RA, the potent reaction of Hpa-tg SF towards TNF-alpha stimulation likely contributed to the severe damage observed in the joints of Hpa-tg mice.

### Increased innate immune-activity in CIA Hpa-tg mice

**Figure 2:**
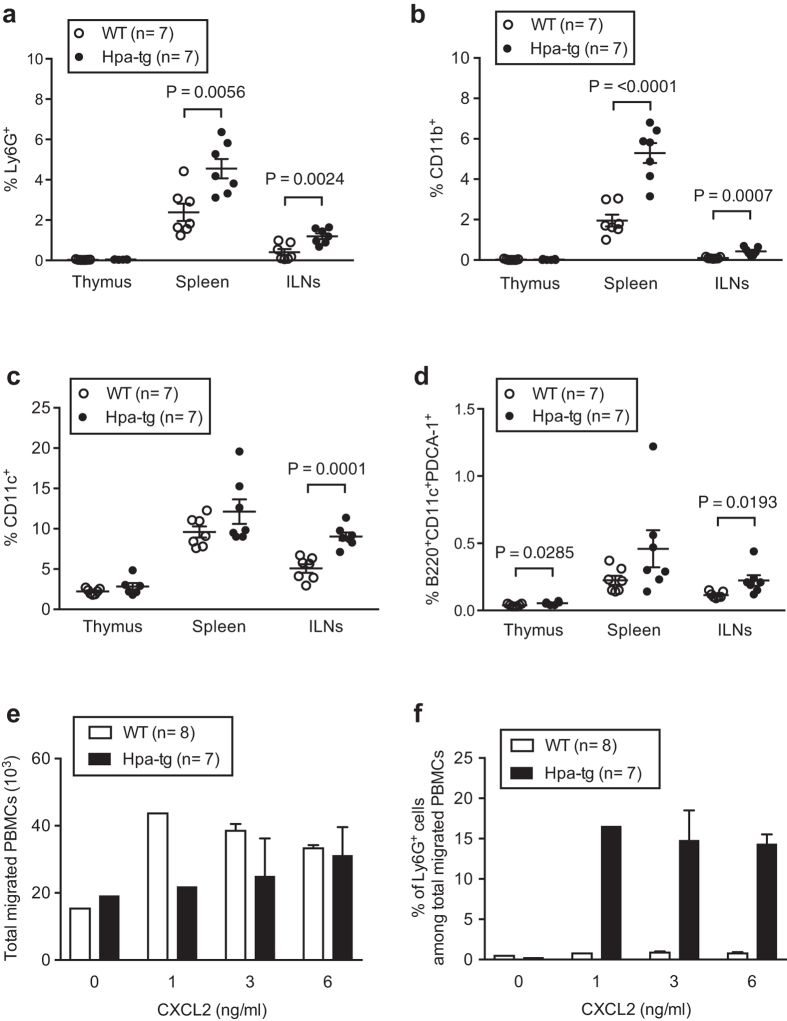
The percentage of innate immune cells is increased in CIA Hpa-tg mice. (**a**–**d**) Total cells were collected from each organ of Hpa-tg and WT mice 7-days after induction and stained with antibodies listed in Supplementary Table S2. The proportions of (**a**) neutrophils (Ly6G^+^), (**b**) macrophages (CD11b^+^), (**c**) antigen presenting cells (CD11c^+^) and (**d**) plasmacytoid dendritic cells (B220^+^CD11c^+^PDCA1^+^) in thymic glands, spleens and ILNs of WT (open circle) and Hpa-tg mice (closed circle) mice was determined by flow cytometer analysis. The results are shown as means ± SEM from two experiments. (**e**,**f**) Total peripheral blood mononuclear cells (PBMCs), isolated from Hpa-tg and WT mice 7-days after induction, were incubated in a trans-well setting with CXCL2 in the lower chamber. The transmigrated cells in the lower chamber were collected after 18 h and stained with the antibodies for FACS counting.

To examine the immune response of the mice, we next analysed cells collected from the thymus, spleen and inguinal lymph nodes (ILNs) of mice 7-days post CIA induction. First, we evaluated neutrophils (Ly6G^+^), macrophages (CD11b^+^), APCs (CD11c^+^) and plasmacytoid dendritic cells (pDCs) (B220^+^CD11c^+^PDCA-1^+^), as these cells play important inflammatory roles during early development of RA^16^,^17^. We found that proportions of Ly6G^+^, CD11b^+^ and CD11c^+^ cells were higher in the spleen and ILNs of Hpa-tg mice (Fig. 2a–c); while the percentage of pDCs were higher in the thymus and ILNs of Hpa-tg mice in comparison to WT mice (Fig. 2d). There were no differences in naïve Hpa-tg and WT mice, suggesting that the increased proportions of the innate immune cells in Hpa-tg mice were a result of CIA induction. Further, we examined the migratory properties of peripheral blood mononuclear cells (PBMCs) isolated from mice 7-days post CIA induction. The result revealed that more WT than Hpa-tg cells migrated at lower concentrations of CXCL2 (Fig. 2e), which is in the line with our earlier findings^7^. However, about 15% of the migrated Hpa-tg PBMCs were Ly6G^+^ cells, compared to about 1.5% Ly6G^+^ cells in WT mice (Fig. 2f). Moreover, the APCs (CD11c^+^) among the Hpa-tg PBMCs also had a higher tendency to migrate compared to WT mice (Supplementary Fig. S3). Altogether, these results indicate that the Hpa-tg mice had a higher activity of innate immunity upon CIA stimulation.

### Increased T-lymphocyte activity in CIA Hpa-tg mice

**Figure 3:**
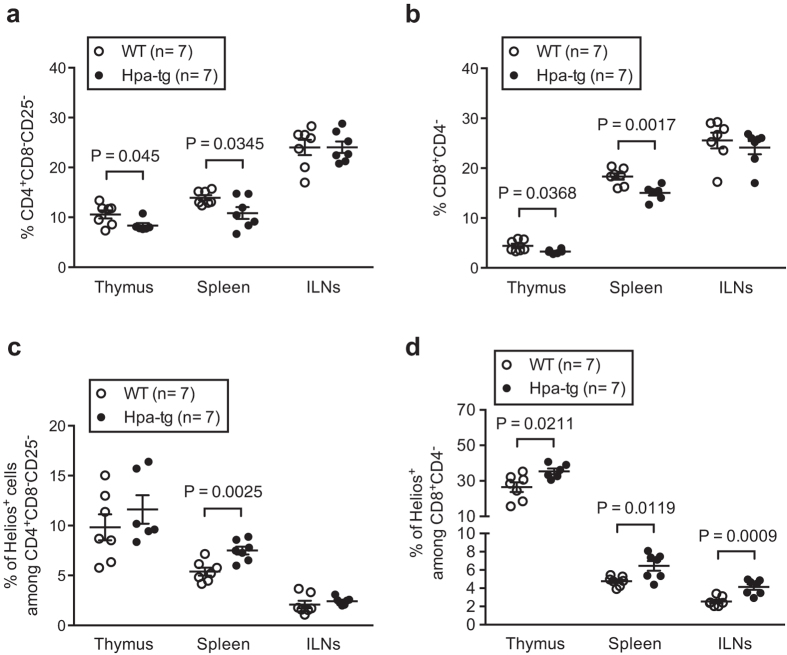
The percentage of Helios^+^ cells among CD4^+^CD25^−^ and CD8^+^ T cells is higher in CIA Hpa-tg mice. The total cells collected from each organ were analysed for (**a**) percentage of CD4^+^CD8^-^CD25^−^ T cells, (**b**) percentage of CD8^+^CD4^−^ T cells; proportions of Helios^+^ cell among (**c**) CD4^+^CD8^−^CD25^−^ and (**d**) CD8^+^CD4^−^ T cells. The results are shown as means ± SEM from two experiments. Gating strategies are shown in Supplementary Fig. S4.

Since CD4^+^CD25^−^ and CD8^+^ T cells play a pivotal role in RA^19^ and CD4^+^CD25^−^Helios^+^ and CD8^+^Helios^+^ T cells have been reported as indicators of T cell proliferation and autoimmune reactivity^20^,^21^, we next analysed the proportions of these cells in thymus, spleen and ILNs of the CIA induced mice. Flow cytometer analysis showed that the total proportion of CD4^+^CD8^−^CD25^−^ and CD8^+^CD4^−^ cells were lower in both the thymus and spleen of the Hpa-tg mice (Fig. 3a,b). In contrast, the proportion of Helios^+^ T cells in the CD4^+^CD8^−^CD25^−^ population was increased in the spleen (Fig. 3c; Supplementary Fig. S4), while the proportion of Helios^+^ T cells in the CD8^+^CD4^−^ population was significantly higher in all three organs of Hpa-tg mice (Fig. 3d; Supplementary Fig. S4).

**Figure 4:**
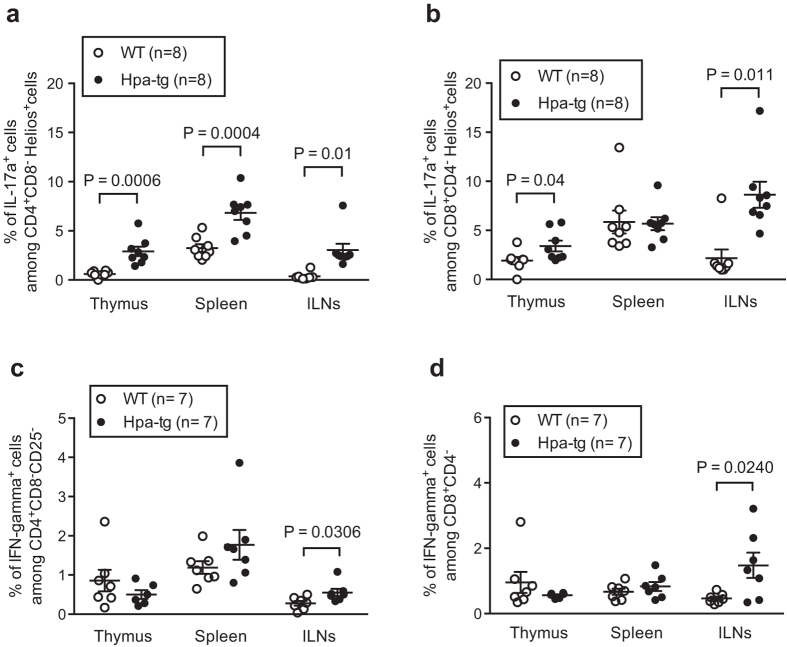
The percentage of IL-17a^+^ and IFN-γ^+^ cells is higher in CIA Hpa-tg mice. The total cells collected from each organ were analysed for proportions of IL-17a^+^ among (**a**) CD4^+^CD8^−^Helios^+^ cells and among (**b**) CD8^+^CD4^−^Helios^+^ cells. The proportion of IFN-γ^+^ cells among CD4^+^CD8^−^CD25^−^ and among CD8^+^CD4^−^ T cells were shown in (**c)** and **(d**), respectively. The results are shown as means ± SEM. Gating strategies are shown in Supplementary Fig. S5.

Subsequently, we determined the proportions of proinflammatory cytokine IL-17a^+^ cells among CD4^+^CD8^−^Helios^+^ and CD8^+^CD4^−^Helios^+^ T cells. The proportions of IL-17a^+^ cells among CD4^+^Helios^+^ T cells were higher in thymic glands, spleens and ILNs of Hpa-tg mice (Fig. 4a). In addition, the proportions of IL-17a^+^ cells were higher among CD8^+^Helios^+^ cells in both thymic glands and ILNs of Hpa-tg mice (Fig. 4b).

The reduction of CD4^+^CD25^−^ and CD8^+^ T cells in the thymus and spleen of Hpa-tg mice could reflect an immune protective effect in Hpa-tg mice. To investigate this, we examined proportions of IFN-γ^+^ T cells, as this pro-inflammatory cytokine was shown to induce inflammation in RA^16^,^17^. We found that the proportion of IFN-γ^+^ cells among CD4^+^CD8^−^CD25^−^ and CD8^+^CD4^−^ T cells was higher in ILNs of Hpa-tg mice (Fig. 4c,d; Supplementary Fig. S5).

**Figure 5:**
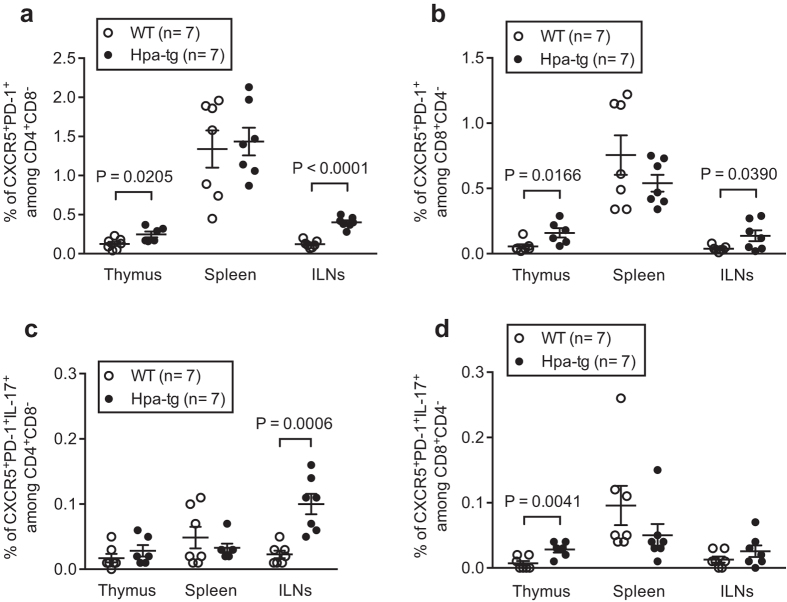
The percentage of Tfh cells among CD4^+^CD25^−^ and CD8^+^ T cells is higher in the Thymus and ILNs of Hpa-tg mice. The total cells collected from each organ were analysed for the proportion of CXCR5^+^PD-1^+^ cells among (**a**) CD4^+^CD8^−^ cells and among (**b**) CD8^+^CD4^−^ cells. The proportions of CXCR5^+^PD-1^+^IL-17a^+^ cells among CD4^+^CD8^-^ and among CD8^+^CD4^−^ were shown in (**c** and **d**) respectively. The results are shown as means ± SEM from two experiments. Gating strategies are shown in Supplementary Fig. S6.

Follicular helper T (Tfh) cells are subsets of CD4^+^ T cells and CD8^+^ T cells, which could be further characterized as CD4^+^CXCR5^+^PD-1^+^ and CD8^+^CXCR5^+^PD-1^+^ Tfh cells^22^. To affirm the activity of T cells we determined the proportion of Tfh cells. We found that the proportions of CXCR5^+^PD-1^+^ Tfh cells were significantly higher among CD4^+^CD8^−^ and CD8^+^CD4^−^ T cells in the thymus and ILNs of Hpa-tg mice (Fig. 5a,b). The proportion of CXCR5^+^PD-1^+^ IL-17a^+^ among CD4^+^CD8^−^ T cells was significantly higher in the ILNs of Hpa-tg mice (Fig. 5c; Supplementary Fig. S6); while the proportion of CXCR5^+^PD-1^+^ IL-17a^+^ among CD8^+^CD4^−^ T cells was higher in the thymus of Hpa-tg mice (Fig. 5d; Supplementary Fig. S6).

**Figure 6:**
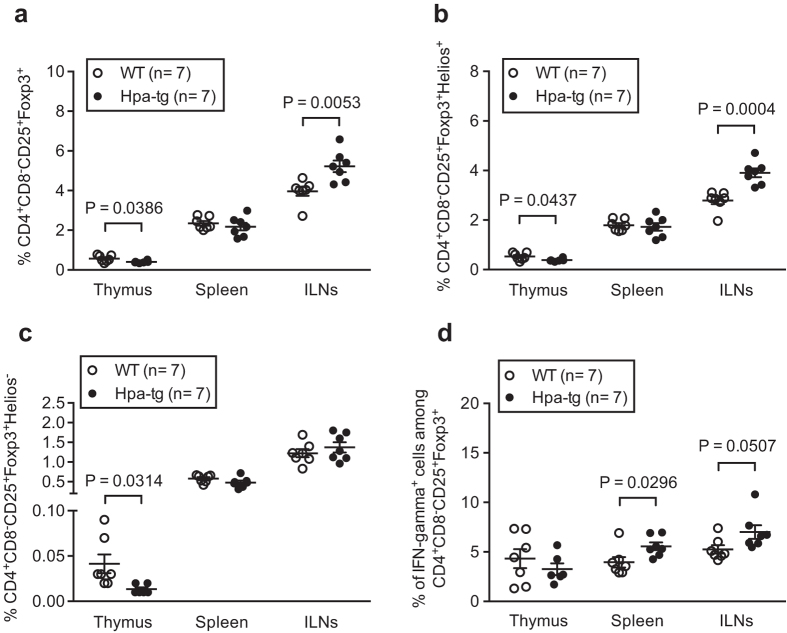
Treg cells switch their phenotype in CIA treated Hpa-tg mice. The proportions of (**a**) CD4^+^CD8^−^CD25^+^Foxp3^+^ Treg cells, (**b**) CD4^+^CD8^−^CD25^+^Foxp3^+^Helios^+^ tTreg cells, (**c**) CD4^+^CD8^−^CD25^+^Foxp3^+^Helios^−^ pTreg cells and the proportion of IFN-γ^+^ cells among (**d**) CD4^+^CD8^−^CD25^+^Foxp3^+^ Treg cells were determined in thymic glands, spleens and ILNs of CIA treated WT (open circle) and Hpa-tg (closed circle) mice by using flow cytometer. The results are shown as means ± SEM from two experiments. Gating strategies are shown in Supplementary Fig. S7.

Regulatory T (Treg) cells drive immune tolerance, helping to protect against development of autoimmune diseases^23^,^24^. To assess the population of Treg cells, we used surface markers CD4 and CD25 together with the intracellular transcription factor Foxp3^25^,^26^,^27^. Treg cells can be further sub-classified as thymus-derived (tTreg) and peripheral-induced (pTreg) cells by either Helios or Neuropilin-1 expression, respectively^28^,^29^,^30^. Herein, we have used Helios as a marker for determining the tTreg cells since we have found that Helios is a more suitable marker in this context^31^. The Hpa-tg mice had an overall lower proportion in thymus of CD4^+^CD25^+^Foxp3^+^ (Fig. 6a), CD4^+^CD25^+^Foxp3^+^Helios^+^ (Fig. 6b) and CD4^+^CD25^+^Foxp3^+^Helios^−^ (Fig. 6c) T cells, which is well in line with the more severe pathology of RA in the Hpa-tg mice. Unexpectedly, the proportions of these T cells were significantly higher in ILNs (except for CD4^+^CD25^+^Foxp3^+^Helios^−^). However, further analysis revealed higher proportions of pro-inflammatory IFN-γ^+^ Treg cells in the spleen and ILNs of Hpa-tg mice (Fig. 6d; Supplementary Fig. S7), suggesting a phenotypic shift of the Treg cells under inflammatory conditions, as observed earlier in a mouse model of type I diabetes^32^ and in RA^33^,^34^.

### Increased numbers of inflammatory cells in the ILN and spleen

**Figure 7:**
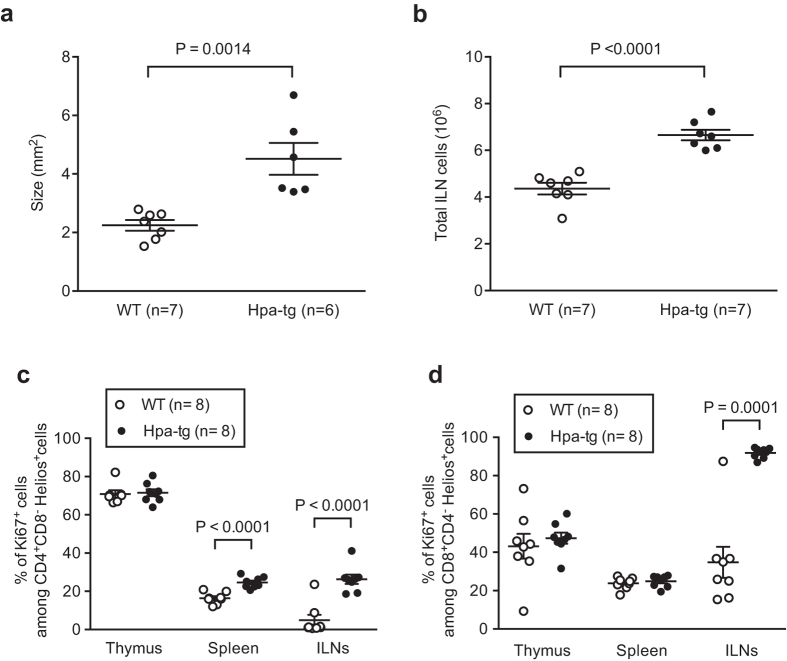
Larger ILNs in CIA treated Hpa-tg mice. (**a**) Size of collected right and left ILNs from collagen-induced WT (n = 7; open circle) and Hpa-tg (n = 6; closed circle) mice were examined by area measurement (Image J). Each dot in the graph represents the average area of both ILNs from one mouse. (**b**) Total cell number of both right and left ILNs from collagen-induced WT (n = 7; open circle) and Hpa-tg (n = 7; closed circle) mice. The proportion of Ki67^+^ cells among (**c**) CD4^+^CD8^−^ Helios^+^ cells and among (**d**) CD8^+^CD4^−^ Helios^+^ cells. The results are shown as means ± SEM.

As ILNs of Hpa-tg mice displayed overall higher proportions of innate immune cells and inflammatory T-cells, it indicates an important role of this immune organ in the pathogenesis of CIA in Hpa-tg mice. Further examination of the ILNs from mice revealed a significantly enlarged size of the organ in Hpa-tg mice, 7 days after CIA induction (Fig. 7a), and a significantly higher number of total cells, suggesting an increased infiltration or proliferation of cells (Fig. 7b). This is also evidenced by immunostaining of ILNs that shows massive infiltration of T cells in the germinal centres of ILNs of Hpa-tg mice (Supplementary Fig. S8). Further, we found that the proportions of Ki-67^+^ cells among CD4^+^CD8^−^Helios^+^ T cells was significantly higher in the ILNs (as well as in the spleen) of Hpa-tg mice (Fig. 7c) and the proportion of Ki-67^+^ cells among CD8^+^CD4^−^Helios^+^ T cells was higher in the ILNs of Hpa-tg mice (Fig. 7d). The proportions of total Ki-67^+^ cells^+^ were also found significantly higher in spleens and ILNs of Hpa-tg mice, but did not differ in thymic glands, compared to WT mice (Supplementary Fig. S9). Taken together, these data illustrates that T cells and other immune cells of Hpa-tg mice have higher proliferation properties than that of WT mice.

To evaluate the extravasation potential of the immune cells, we analyzed the proportion of CCR6^+^ cells among T cells, APCs and Ly6G^+^ neutrophils, since it has been shown that a variant of CCR6^+^ cells is associated with RA^35^. The proportions of CCR6^+^ cells among CD4^+^CD8^−^ T cells and CD11c^+^ APCs were higher in thymic glands, but were lower in the spleens and ILNs of Hpa-tg mice (Supplementary Fig. 10a,c). The proportions of CCR6^+^ cells among CD8^+^CD4^−^ T cells and Ly6G^+^ cells were significantly elevated in thymic glands of Hpa-tg mice (Supplementary Fig. 10b,d). Overall, the proportion of CCR6^+^ cells is higher in the thymus, but lower in the spleen and ILNs. Since the thymus is the location for T cell maturation, increased CCR6 expression 7 days after CIA could be indicative of the severity in the following phase of CIA with joint pathology.

### Enhanced response of Hpa-tg splenic T cells

**Figure 8:**
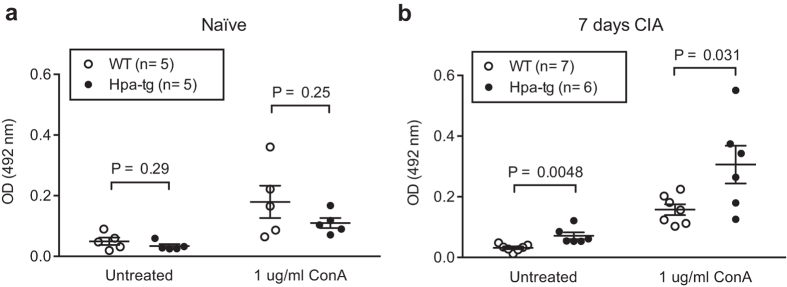
Increased proliferation of splenic T lymphocytes from CIA treated Hpa-tg mice. Splenocytes were isolated from spleen of (**a**) naïve mice and (**b**) mice 7 days post CIA. The cells were cultured in 96-well plates with or without addition of Con A for 60 h. Proliferation was determined using the MTS assay. The data are mean value of triplicates. n = 5 mice in naïve groups; n = 7 in CIA WT mice; n = 6 in CIA Hpa-tg mice.

Finally, we examined proliferation of splenic T lymphocytes from mice (naïve or 7 days post CIA induction). The primary cultured splenic cells isolated from naïve Hpa-tg and WT mice showed a similar proliferation rate under the culture conditions. Con A stimulation promoted proliferation of both cell groups to a similar level (Fig. 8a). However, splenic lymphocytes from Hpa-tg mice 7-days post CIA induction displayed significantly higher proliferation rates than WT cells, even without Con A stimulation (Fig. 8b). This indicates that overexpression of heparanase has amplified the inflammatory reaction of splenic cells in response to CIA stimulation.

## Discussion

HSPGs participate in inflammatory reactions through several pathways^2^,^36^. Accumulated evidence showed that HS binding to chemokines facilitates formation of gradients that are necessary for directed cell migration. This interaction depends on the molecular structure of HS that is strictly regulated in the process of biosynthesis and modulated by post-synthesis enzymes, such as heparanase^37^. Several earlier reports have demonstrated the important roles of heparanase in leukocyte transmigration^7^ and macrophage activation^8^,^38^,^39^,^40^. The observation that heparanase level was 100-fold higher in the synovial fluid of rheumatoid arthritis (RA) patients^12^ prompted us to investigate the impact of heparanase by examining the immunological responses in a murine model of RA.

The chicken collagen induced arthritis (CIA) model is customized to give relatively higher incidence in the C57Bl/6 mice^18^. Our experiments agreed with this notion and the results exhibited a similar pattern in the incidence of diseases between the two groups of Hpa-tg and WT mice. The clinical scores correlated well with the histological examinations, *e.g.* the joint of the mouse with the highest scores displayed more infiltration of inflammatory cells and bone destruction. Furthermore, the infiltration of cells into the joints was associated with increased proportions of innate immune cells in the ILN and the spleen, since the proportions of neutrophils (Ly6G), macrophages (CD11b) and antigen-presenting dendritic cells (CD11c) were higher in the immune organs of Hpa-tg mice. The significantly higher proportion of neutrophil migration towards chemoattractant CXCL2 also supports this conclusion. It is known that inflammation in the joints activates resident synovial fibroblasts (SF), manifesting arthritic pathology^41^. The higher mean stimulation index of SFs from Hpa-tg mice upon TNF-alpha stimulation suggests that heparanase expression enhanced the cellular response to inflammatory stimuli. Thus, it can be proposed that increased levels of heparanase in SF of RA patients may promote SF proliferation and infiltration into the joints.

Accumulating reports suggest that RA is primarily a T lymphocytes-mediated autoimmune disease, as T cells are one of the most abundant cell types in RA synovium^1^,^42^,^43^. To find out whether heparanase expression affect T cell activity, we analyzed T cells from lymphoid organs including thymus, spleen and inguinal lymph nodes (ILNs) dissected from mice 7-days post CIA. Collectively, we found that the proportions of CD4^+^CD8^−^CD25^−^ and CD8^+^CD4^−^ T cells were lower in the thymus and spleen of Hpa-tg mice vs. WT mice. However, it should be noted that the proportion of Helios^+^ cells among CD4^+^CD8^−^CD25^−^ was higher in the spleen and among CD8^+^CD4^−^ T cells was higher in all three organs of Hpa-tg mice. This suggests a higher auto-reactivity of T cells^20^,^21^, which most likely contributed to the higher clinical scores of RA in Hpa-tg mice. Moreover, the proportions of Th1, Tfh and IL-17^+^ Tfh cells were higher in Hpa-tg mice, further supporting our findings given that these cells play pivotal roles in RA^44^,^45^. Though the proportions of T cells in ILNs did not significantly differ between Hpa-tg and WT mice, the proportion of Helios^+^ cells among CD8^+^CD4^−^ T cells was higher in Hpa-tg mice. Furthermore, the proportion of Ki-67^+^ cells among CD4^+^CD8^−^Helios^+^ and CD8^+^CD4^−^Helios^+^ cells were higher in ILNs of Hpa-tg mice, which indicates that the T cells of Hpa-tg mice had higher proliferation property. In addition, the proportions of RA-associated IL-17a-expressing cells among Helios^+^ CD4^+^ and CD8^+^ T cells were also higher in thymic glands and ILNs of Hpa-tg mice, as well as in the spleen among CD4^+^ T cells. As the draining LN, ILNs represent a localized immune response, presenting antigens. Thus, one could expect to have an early immune response in ILNs after CIA. This reaction was stronger in the Hpa-tg mice, revealed by the enlarged size of the ILNs that had more cells due to an increased infiltration and proliferation, which may have led to the early appearance of symptoms in Hpa-tg mice.

Naturally occurring CD4^+^CD25^+^Foxp3^+^regulatory T (Treg) cells maintain immunological self-tolerance and prevent a variety of autoimmune diseases, including RA^46^,^47^. It has been shown that re-establishing the T-cell balance in favour of Treg cells in animal models of rheumatic disease attenuated autoimmune responses^34^. The higher proportion of Treg cells (both tTreg and pTreg cells) in the ILNs of Hpa-tg mice was likely a result of higher activity of pDCs^48^. Nevertheless, this increased proportion of Treg cells failed to recruit a protective action against autoimmunity. Earlier studies have found that Treg cells become pro-inflammatory T effector cells under pathological conditions such as type I diabetes and RA^32^,^33^,^34^. The increased proportions of IFN-γ^+^ cells among Treg cells in the spleen and ILNs of Hpa-tg mice also suggest a phenotypic shift of Treg cells in Hpa-tg mice. On the other hand, differential change in the number of Treg cells in the immune organs upon stimulation has been observed^49^. As the Treg cells were modulated by granulocyte macrophage colony-stimulation factor (GM-CSF)-stimulated DCs^50^, examination of the cytokine activity in Hpa-tg mice may provide further information.

It has been demonstrated that heparanase expression is critical for T cell infiltration into tumor tissues^51^. Our findings of increased number of Helios^+^ T cells, Th1, Tfh and IL-17^+^ Tfh cells in Hpa-tg mice supports the functional roles of heparanase for T cell activity in diseases. In line with this hypothesis we found a higher migration property of Ly6G+ cells. Along with our recent finding that heparanase expression activates macrophages^40^, the functional roles of heparanase in immune reactivity should be further investigated in diseases apart from RA. Further, heparanase degradation of HS may also have contributed to the inflammatory cell migration. An earlier study demonstrated that overexpression of heparanase in mice remodelled the vascular basement membrane and increased vessel permeability, leading to augmented delayed-type hypersensitivity^52^. Thus, it seems that heparanase expression confronted the potential tolerance of animals upon CIA induction^53^.

In conclusion, our results demonstrate a harmful effect of heparanase expression in a murine RA model, which may reflect the situation of RA patients^12^. The data suggest that elevated levels of heparanase enhance the immune response of the mice, specifically the activity of T lymphocytes. The severe pathological symptoms of Hpa-tg mice were also accompanied by increased proliferation rates of synovial fibroblasts under stimulation with TNF-alpha. This is highly relevant to RA where TNF-alpha is a major player in the pathogeneses of inflammatory reactions^54^. Thus, our ongoing studies on testing the effect of heparanase inhibitors will hopefully provide information for development of novel treatment for RA patients.

## Methods

### Mice

Heparanase transgenic mice (Hpa-tg) were generated as described^15^ and crossed to a C57Bl/6 background for more than 10 generations. Wildtype C57Bl/6 (WT) mice were used as control. The mice were maintained at the animal facility of Biomedical Center (Uppsala University) with free access to water and chow. The animal experiments were performed in accordance with Swedish regulations and guidelines (Swedish Board of Agriculture: SJVFS 2012:26) and approved by the local animal ethics committee (Uppsala).

### Collagen-induced arthritis (CIA)

CIA was applied essentially as described^18^. Briefly, collagen from chicken sternal cartilage (Sigma) was dissolved in 0.1 M acetic acid overnight on a shaker at 4 °C to a final concentration of 2 mg/mL. The collagen solution was then emulsified 1:1 with Complete Freund’s Adjuvant (Sigma), containing 1 mg/ml heat-inactivated *M.tuberculosis,* using a homogenizer in an ice bath. The freshly made antigen emulsion, containing 200 μg of collagen and 50 μg of inactivated *M.tuberculosis*, was injected subcutaneously to each mouse at the base of the tail with a 27-gauge needle. Mice were boosted with an identical injection 21 days later. Twenty days post the initial induction, the mice were examined 3 times/week for scoring clinical symptoms (joint swelling) by visual inspection^55^. Swelling degree of wrist/ankle and palm was each credited with 5 points, while each swollen toe digit was credited with 1 point. The maximum score for an individual mouse was therefore 60 points. The experimental mice were coded and blindly examined by two experienced researchers for the clinical scoring of inflammation. Animals sacrificed early, due to suffering or high arthritic score, were included in the statistics with their last known score.

### Sample collection and preparation

The endpoint was set to either when the animals showed signs of suffering, a full swelling score (60) or a maximum of 85 days post first stimulation. Upon sacrifice, the front and hind limbs were excised and fixed in 4% paraformaldehyde phosphate buffer for 24 h at room temperature. The immune organs were collected after sacrificing the mice of naïve, day 7 post CIA induction and at the end point.

### Histological analysis

The fixed limbs were decalcified in Parengy solution (0.15% chromethrioxid, 4.3% nitric acid, and 30% ethanol; Bie & Berntsen A-S, Copenhagen, Denmark) for 8–12 days at room temperature, and paraffin-embedded. The limbs embedded in paraffin were sectioned in sagittal. The sections were stained with haematoxylin/eosin. The pathological parameters (bone erosion and cell infiltration) were graded from 0 to 3 as follows: 0, absent; 1, minor changes; 2, moderate; 3, severe, by blind analysis. Scores reflect the mean value of the overall grade of all affected joints in 1 selected limb per mouse.

### Flow cytometry analysis

Thymic glands, spleens and inguinal lymph nodes (ILNs) were harvested from Hpa-tg and WT mice 7-days post CIA injection (n = 7). Single cell suspensions from these organs were prepared as described earlier^31^ and stained with surface markers: CD4, CD8, CD25, CXCR5, PD-1, CD19, B220, Ly6G, CD11c, PDCA-1, CXCR6 and CD11b (Supplementary Table S2). The cells were then fixed and permeabilised with Fixation Permeabilisation buffer (eBioscience) for staining of intracellular markers: Foxp3, Helios, IFN-γ, Ki-67 and IL-17a (Supplementary Table S2). The samples were analysed on LSRFortesa (BD) using DivaDacker software (BD). One million events were counted for each sample and the fluorescence minus one and single stained controls were used for gating strategies as described earlier^31^. The FCS files were analysed on FlowLogic software (Inivai Technologies).

### Lymph node analysis

The size of collected right and left ILNs from CIA treated WT (n = 7) and Hpa-tg mice (n = 6) were examined by area measurement using Image J software. The average area of left and right ILN from each mouse was attained by normalization to the area of a well of a 24-well plate. The total number of cells from ILNs was determined using an LSRFortessa flow cytometer (BD). For histologic analysis 5–7 um sections were cut and fixed in acetone for 10 minutes, and subsequently incubated with relevant antibodies at room temperature for 45 minutes, and when needed, further incubated with appropriate secondary antibodies or reagents for 30 minutes. Antibodies used were: AlexaFluor 488 labelled anti-mouse CD3 (clone 145–2C11, eBioscience Inc.), unlabelled anti-mouse IgD (clone 11–26c, Southern Biotech) and AlexaFluor-647 labelled anti-CR1 (clone 8C12). Unlabelled rat antibody was detected by AlexaFluor 555 labelled anti-rat IgG (Invitrogen).

### Splenocyte proliferation assay

Splenic lymphocytes were isolated from mice of naïve or 7 days post CIA induction essentially as described^56^. Briefly, after sacrificing the mice, individual spleens were excised and cells were separated with a 100 μm strainer. Erythrocytes were lysed with low osmotic Gey’s solution followed by three separate washing steps with cold PBS. The splenocytes were seeded in triplicates at the same density (2 × 10^4^ viable cells) into 96-well plates with or without addition of 1μg/ml Concanavalin A (Con A; Sigma C5275) and cultured in DMEM supplemented with 10% fetal bovine serum and 100 U/ml of penicillin-streptomycin. After 60 h of culture at 37 °C, cell proliferation was analysed using the CellTiter 96 AQueous One Solution Cell Proliferation Assay (MTS) (Promega, Madison, WI, USA), according to the manufacturer’s instruction.

### Synovial fibroblast proliferation assay

Synovial fibroblasts (SFB; VCAM^+^ confirmed by Western blot analysis) were isolated from naïve Hpa-tg (n = 5) and WT (n = 5) mice essentially as described^57^. Briefly, after euthanasia of the animals with CO_2_, skin was removed and the hind feet of the mice were isolated distal of the tibia. The ankles were then separated by micro-dissection and digested with collagenase type IV (Sigma C5138) for 1 h at 37 °C. The resulting cell suspension was cultured in DMEM supplemented with 10% fetal bovine serum and 100 U/ml of penicillin-streptomycin. Cells between passage 4 and 6 were subjected to TNFα (10 ng/ml) stimulation for 48 h. Proliferation of the cells was analysed using the MTS assay as above. Stimulation index was calculated by dividing the OD (492 nm) of stimulated cells with that of the non-stimulated cells from corresponding mouse.

### Western Blotting

Cells were lysed in RIPA buffer (50 mM Tris pH 7.5, 150 mM NaCl, 1% Triton X-100, 1% Sodium Deoxycholate, 1 mM EDTA, 0.1% SDS, 1 mM NaF, 1 mM Na_3_VO_4_) and complete mini protease inhibitor mixture (catalogue no. 04-693-116-001, Roche) and centrifuged at 13,000 rpm, 10 min. The protein concentration of the supernatant was determined by BCA assay. A total of 5 μg of protein was separated on a 12% SDS-PAGE. After blotting to a nitrocellulose membrane, heparanase was detected by incubation with an anti-heparanase antibody (#1453)^58^ followed with an anti-rabbit antibody. The signals were visualized with SuperSignal West Dura Extended Duration Substrate (Thermo Scientific).

### Transwell migration assay

The migration of peripheral (Ly6G^+^ cells) and APCs (CD11c^+^ cells) were assessed using a 24-Transwells, 8.0 μm pore size and polycarbonate treated polystyrene plate (Corning Incorporated, Costar, USA). The peripheral blood mononuclear cells (PBMC) (2 × 10^6^) were seeded on the upper chamber. The RPMI-1640 supplemented with FBS and antibiotics were added in the lower chamber with or without recombinant murine MIP-2 (CXCL2) (PeoroTech) at the concentrations indicated. The migration of total PBMC was assessed after 18 h incubation at 37 °C humidified chamber containing 5% CO_2_ by collecting cells from the lower chamber and staining them with antibodies for flow cytometry analysis that was performed by the same way as described above.

### Statistics

Data are expressed as mean values and standard errors of the mean (SEM). The Mann-Whitney U test was used for statistical analysis of clinical and histological scores. Unpaired Student’s t test was used for analysing cell proliferation and flow cytometry analysis. A p value of ≤0.05 was considered statistical significant.

## Additional Information

**How to cite this article**: Digre, A. *et al*. Overexpression of heparanase enhances T lymphocyte activities and intensifies the inflammatory response in a model of murine rheumatoid arthritis. *Sci. Rep.* **7**, 46229; doi: 10.1038/srep46229 (2017).

**Publisher's note:** Springer Nature remains neutral with regard to jurisdictional claims in published maps and institutional affiliations.

## Supplementary Material

Supplementary Figures and Tables
