# Supplementary material for: Overexpression of heparanase enhances T lymphocyte activities and intensifies the inflammatory response in a model of murine rheumatoid arthritis
Source: Sci Rep. 2017 Apr 12;7:46229. doi: 10.1038/srep46229 (PMC5388921; doi:10.1038/srep46229)
Supplement: Supplementary Figures and Tables [file srep46229-s1.pdf]

Supplementary Figures and Tables

Overexpression of heparanase enhances T lymphocyte activities and intensifies the inflammatory response in a model of murine rheumatoid arthritis

Andreas Digre[1#], Kailash Singh[2#], Magnus Åbrink[3], Rogier M. Reijmers[4], Stellan Sandler[2], Israel Vlodavsky[5] and Jin-Ping Li[1]*

[1]Department of Medical Biochemistry and Microbiology, University of Uppsala, The Biomedical Center, Box 582, SE-751 23 Uppsala, Sweden; [2]Department of Medical Cell Biology, Uppsala University, The Biomedical Center, Box 571, SE-751 23; [3]Department of Biomedical Sciences and Veterinary Public Health, Section of Immunology, Swedish University of Agricultural Sciences, VHC, Box 7028, SE-75007 Uppsala, Sweden [4]Department of Molecular Cell Biology and Immunology, VU University Medical Center, Amsterdam, The Netherlands; [5]Cancer and Vascular Biology Research Center, The Bruce Rappaport Faculty of Medicine, Technion, Haifa, Israel;

[#] These authors contributed equally to this work
*To whom correspondence should be addressed: Jin-ping Li (jin-ping.li@imbim.uu.se), Department of Medical Biochemistry and Microbiology, University of Uppsala, Uppsala, Sweden; Tel: 0046184714241

Supplementary Figure S1: Histological analyses of the joint tissues.

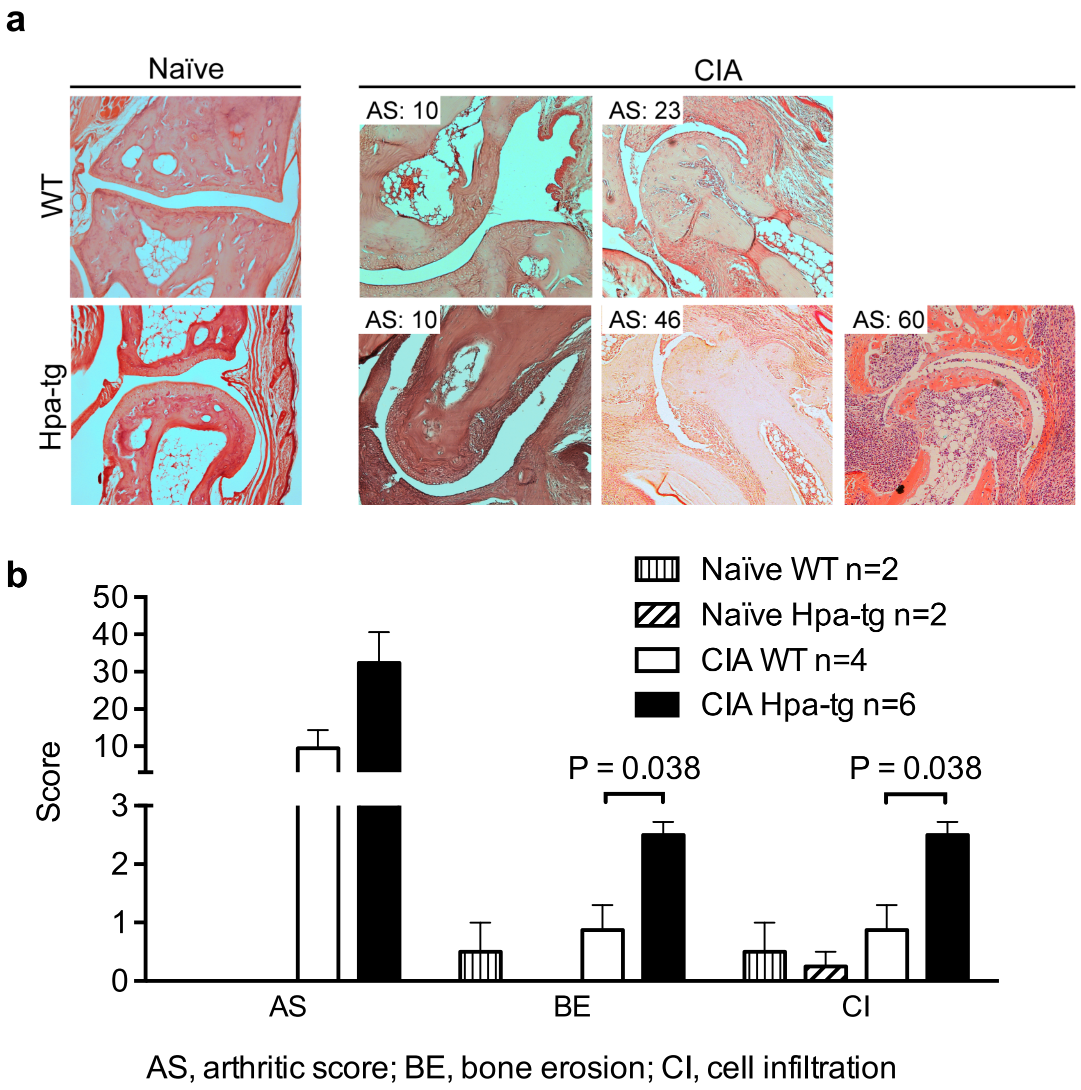

(**a**) Representative H&E stained sections of paw collected from naïve and collagen-induced WT and Hpa-tg mice. (**b**) Semi-quantitative analysis of bone/cartilage erosion (BE) and cell infiltration (CI). The data are mean values of affected joints in all arthritic mice. Data from naïve mice are mean values of all joints in one limb per mouse.

Supplementary Figure S2: Western blot analysis of heparanase expression in primary cultured SF.

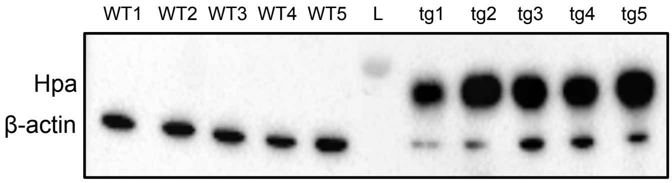

Comparative amounts of total protein (5 μg) from SF cell lysates of each mouse (n=5) from each group were analysed using anti-heparanase antibody (#1453). β-actin was used as loading control. Protein standard ladder (L) band represent 55kDa. (WT: wildtype; tg: Hpa-tg)

Supplementary Figure S3: High proportion of Hpa-tg CD11c^{+} APCs among migrated PBMCs.

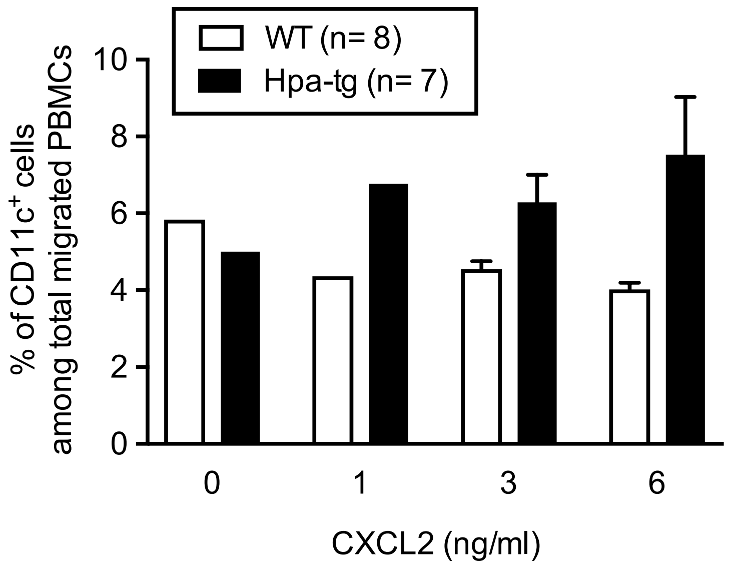

Proportion of CD11c^{+} cells among migrated PBMC, isolated from WT (white bar; n=8) and Hpa-tg (black bar; n=7) mice 7 days post CIA, analyzed by trans-well migration assay at different concentrations of CXCL2 followed by flow cytometer analysis. The results are shown as means±SEM from duplicate (3 and 6 ng/ml CXCL2) or single (0 and 1 ng/ml CXCL2) measurements of pooled PBMCs.

Supplementary Figure S4: Representative histograms of flow cytometer analysis of Helios$^+$ cells among CD4$^+$CD25$^-$ and CD8$^+$ T cells.

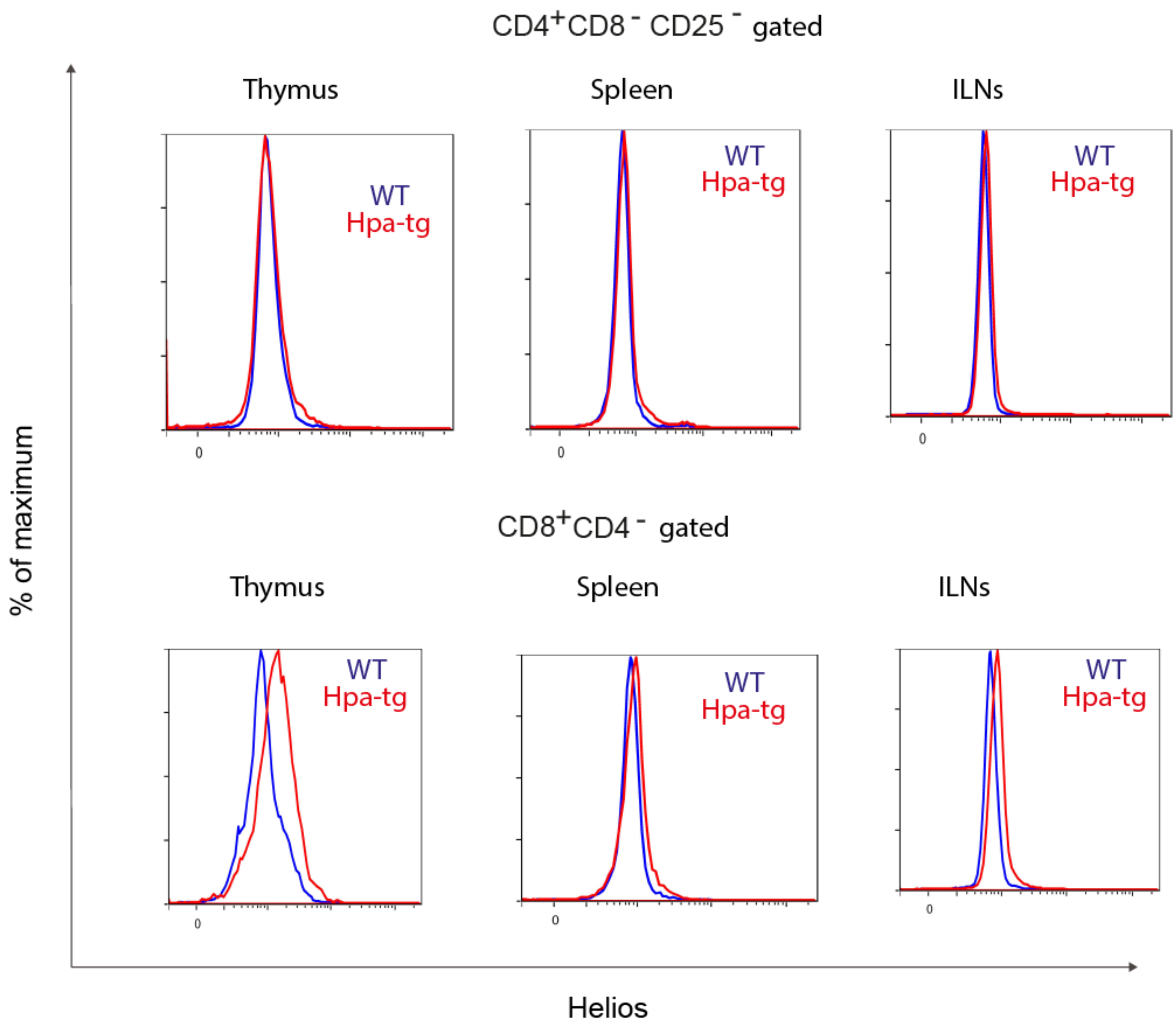

Representative histograms of flow cytometer analysis showing the gating strategy for detection of Helios$^+$ cells among CD4$^+$CD25$^-$ and CD8$^+$ T cells in thymus, spleen and ILNS of CIA WT (blue area) and Hpa-tg (red area) mice.

Supplementary Figure S5: Representative histograms of flow cytometer analysis of IFN-γ^{+} cells among CD4^{+}CD25^{-} and CD8^{+} T cells.

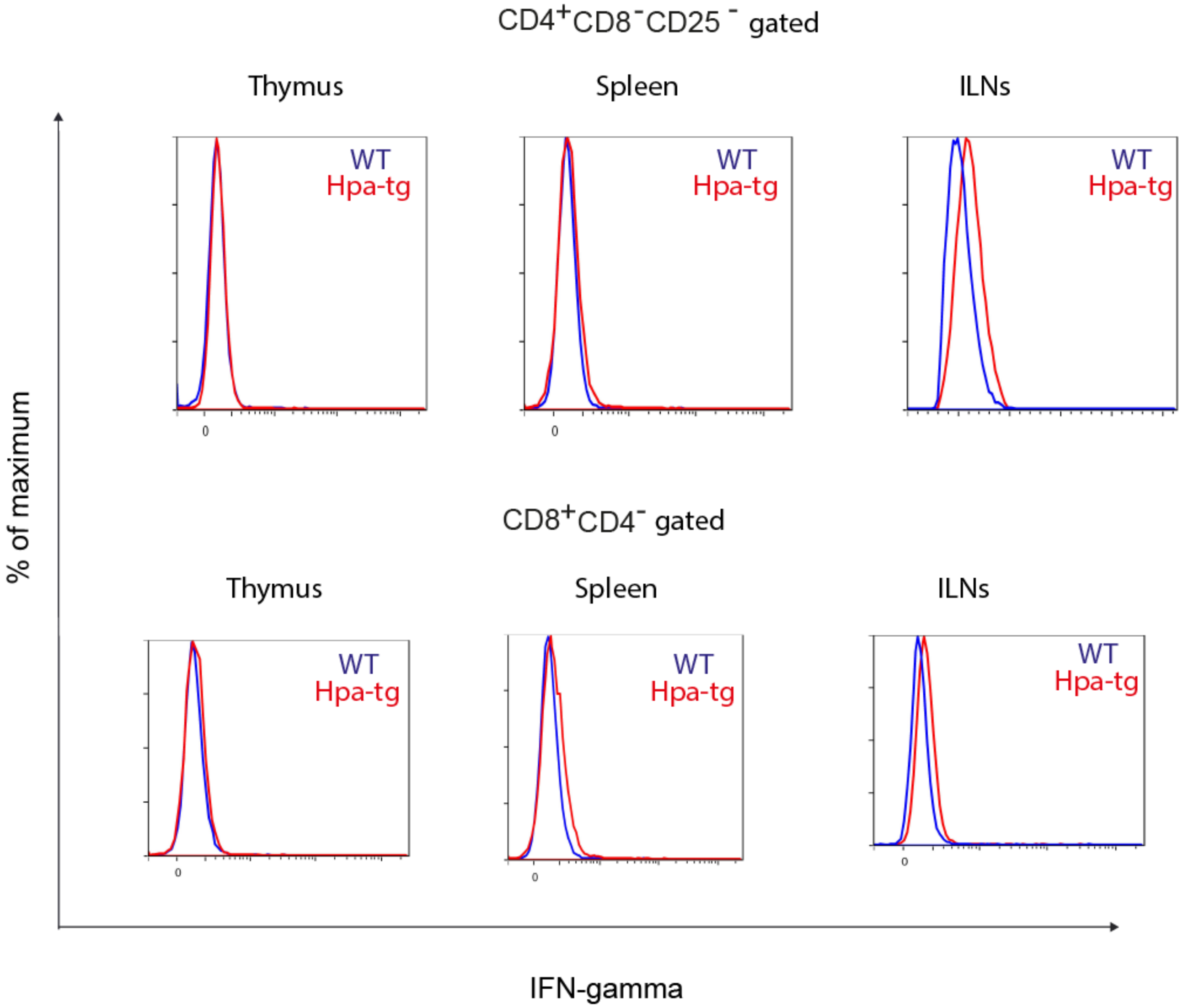

Representative histograms of flow cytometer analysis showing the gating strategy for detection of IFN-γ^{+} cells among CD4^{+}CD25^{-} and CD8^{+} T cells in thymus, spleen and ILNs of CIA WT (blue area) and Hpa-tg (red area) mice.

Supplementary Figure S6: Representative histograms of flow cytometer analysis of IL-17a cells among $CD4^{+}CD8^{-}CXCR5^{+}PD\text{-}1^{+}$ and $CD8^{+}CD4^{-}CXCR5^{+}PD\text{-}1^{+}$ T cells.

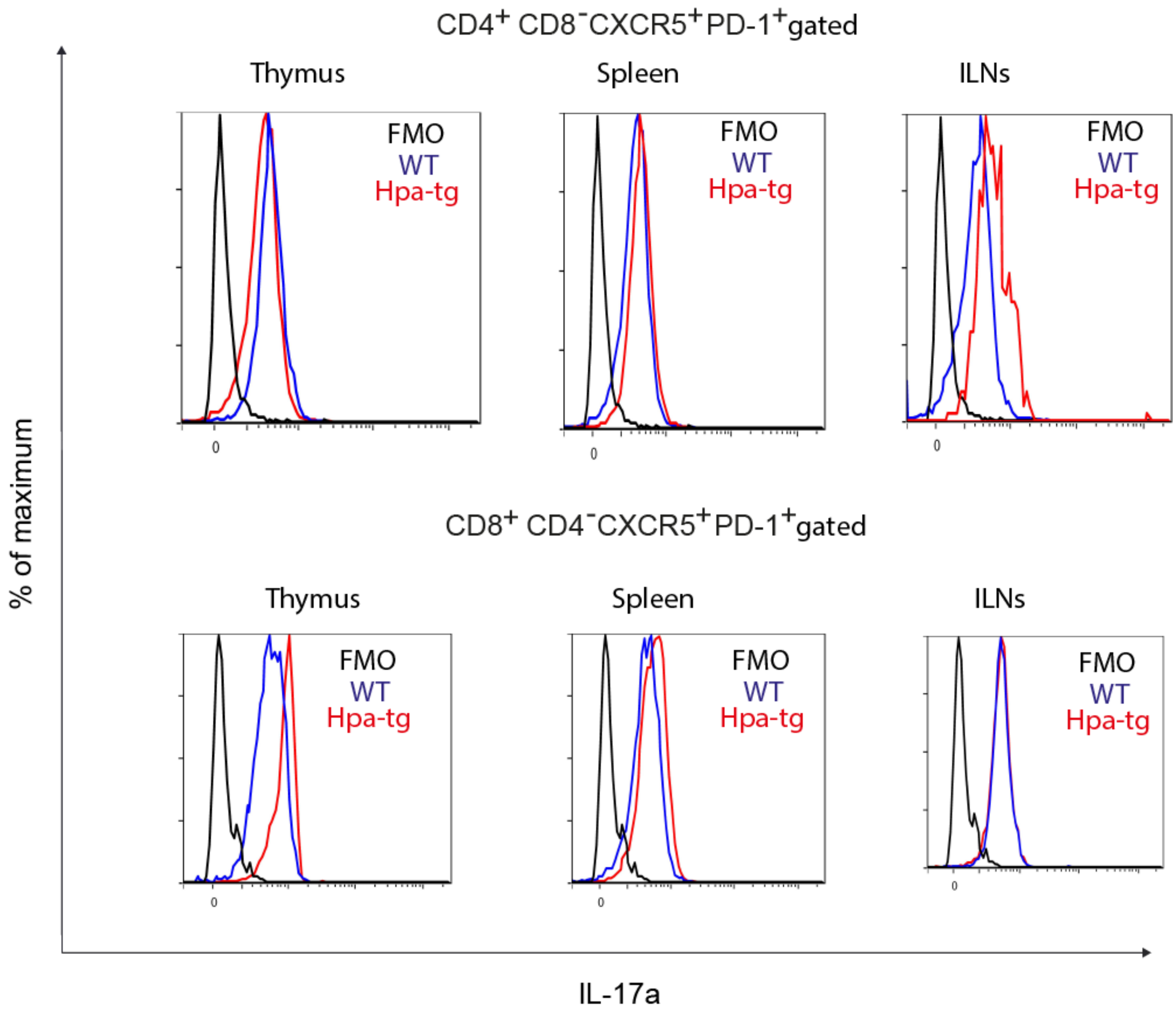

Representative histograms of flow cytometer analysis showing the gating strategy for detection of IL-17a cells among $CD4^{+}CD8^{-}CXCR5^{+}PD\text{-}1^{+}$ and $CD8^{+}CD4^{-}CXCR5^{+}PD\text{-}1^{+}$ T cells in thymus, spleen and ILNs of CIA WT (blue area) and Hpa-tg (red area) mice.

Supplementary Figure S7: Representative histograms of flow cytometer analysis of IFN-γ^+ cells among $CD4^+CD25^+$Foxp3+ Treg cells.

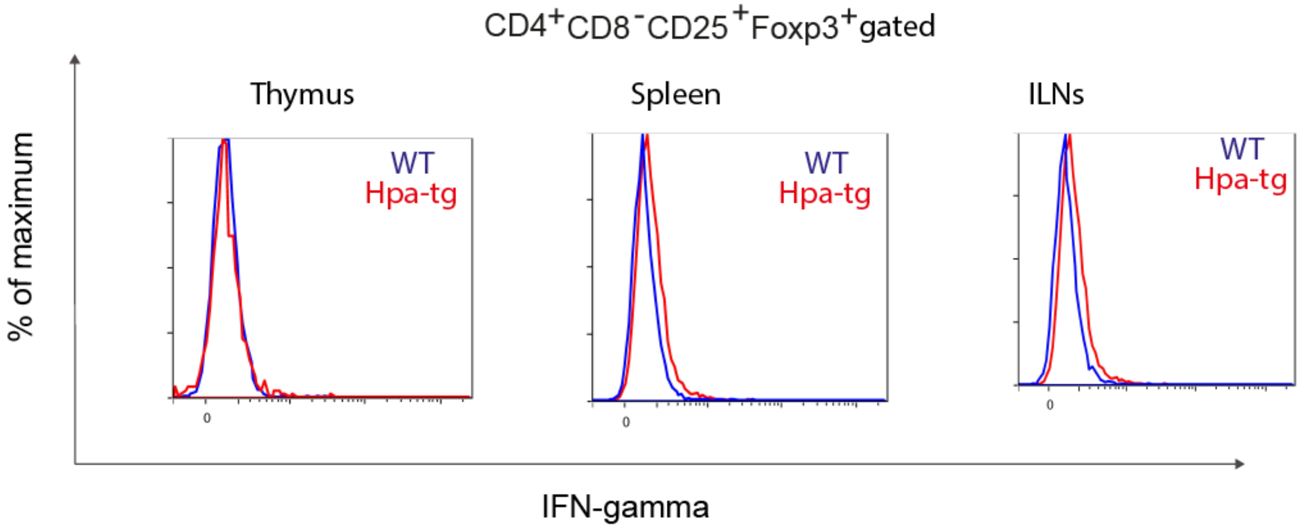

Representative histograms of flow cytometer analysis showing the gating strategy for detection of IFN-γ^+ cells among $CD4^+CD25^+$Foxp3+ Treg cells in thymus, spleen and ILNs of CIA WT (blue area) and Hpa-tg (red area) mice.

Supplementary Figure S8: Increased number of T cells in the germinal center (GC) of end-stage CIA Hpa-tg mouse lymph nodes (LNs).

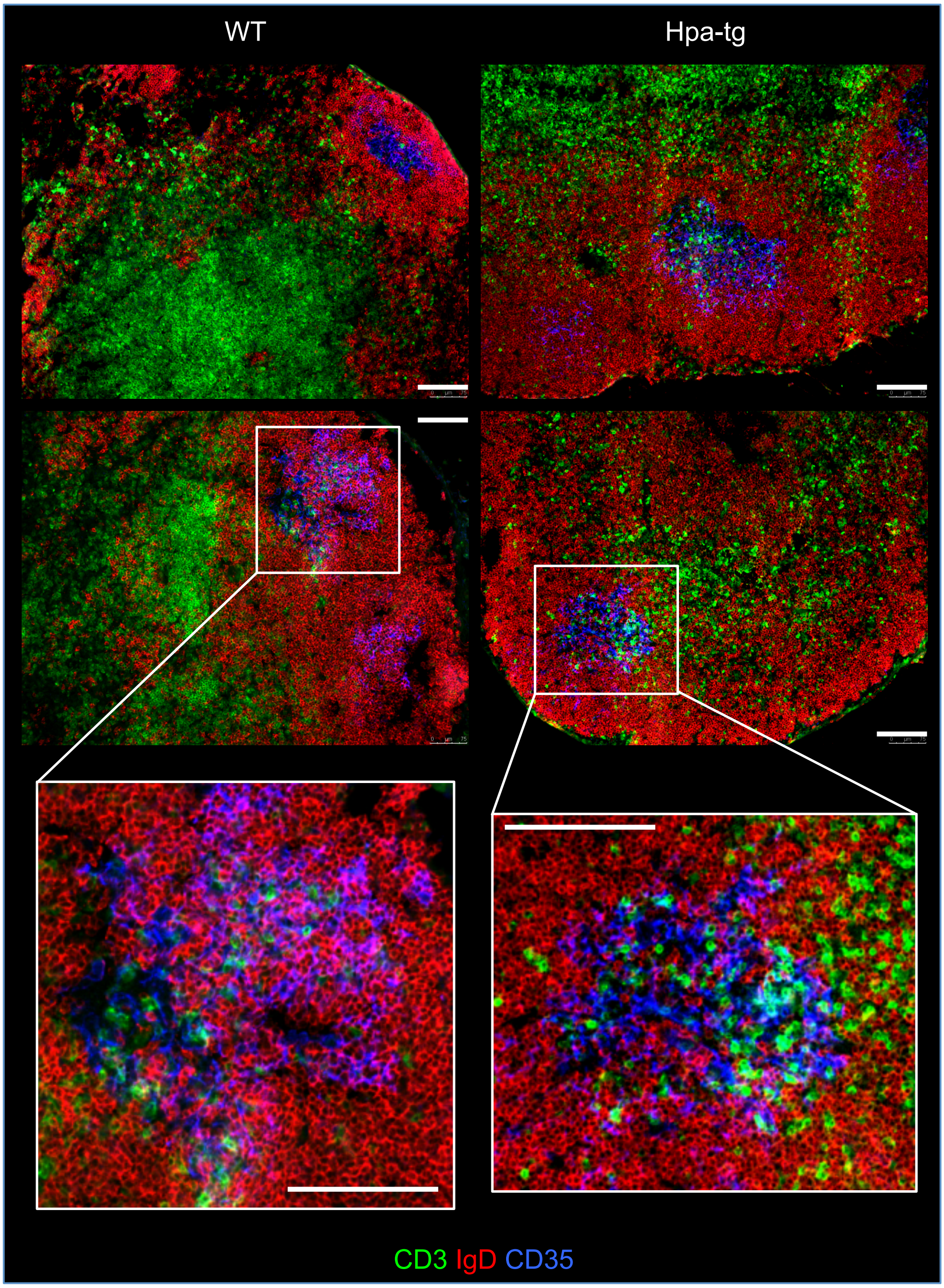

Cellular organization of end-stage CIA WT and Hpa-tg mouse LNs were examined by immunohistological staining of T cells (CD3, green), B cells (IgD, red) and follicular dendritic cells (CD35, blue). Scale bar represent 100 µm.

Supplementary Figure S9: Higher proportions of Ki-67^{+} cells in Hpa-tg mouse spleen and ILNs.

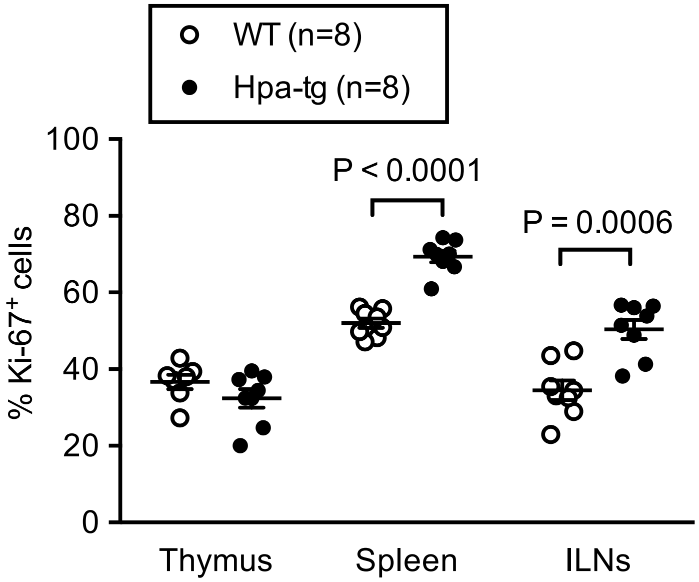

The proportion of Ki-67^{+} cells in immune organs from WT (open circle) and Hpa-tg (closed circle) mice is shown. The results are shown as means±SEM (n=8 in each group).

Supplementary Figure S10: Proportions of CCR6$^+$ cells among CD4$^+$ and CD8$^+$ T cells, CD11c$^+$ APCs and Ly6G$^+$ neutrophils.

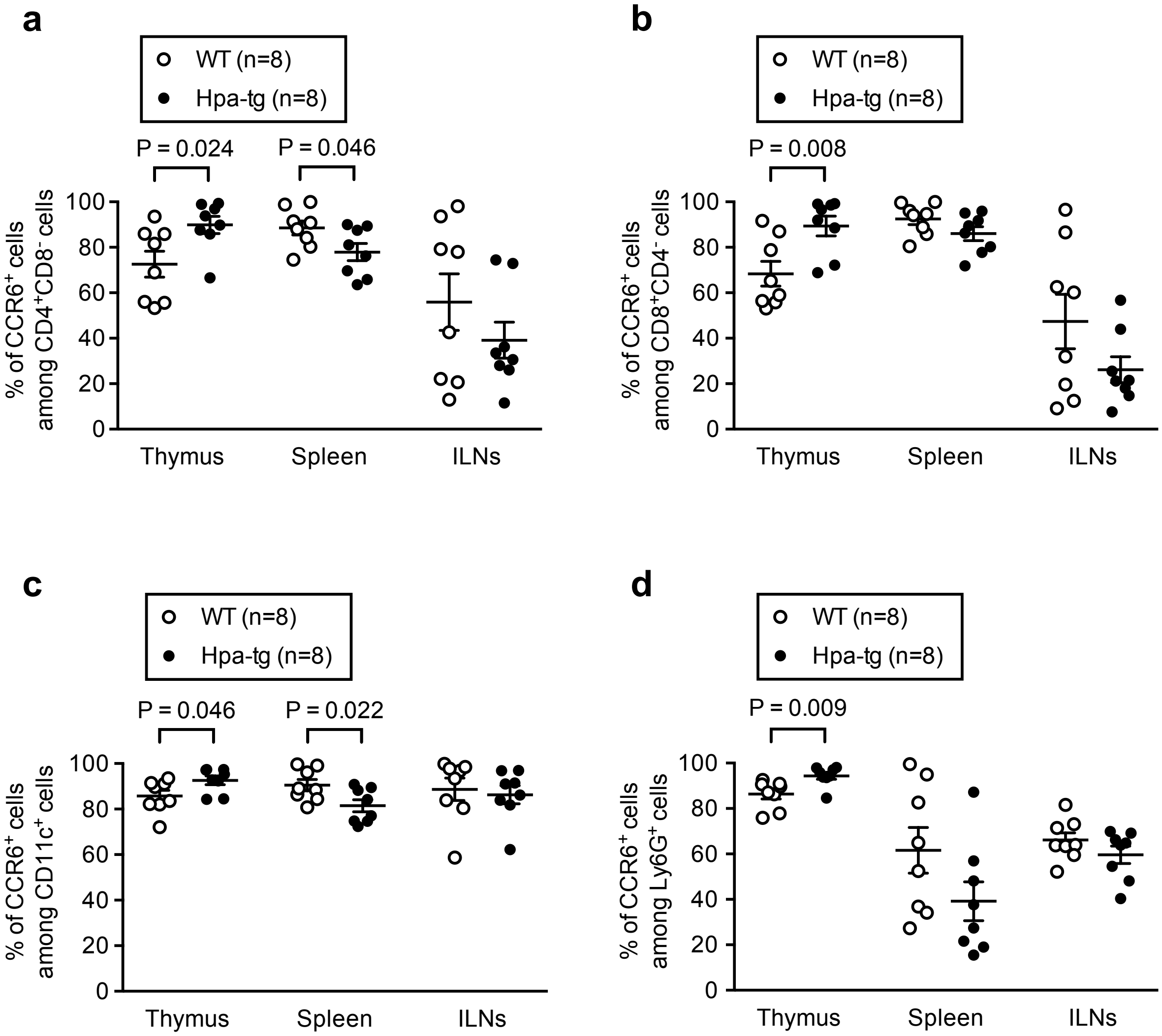

The proportion of CCR6$^+$ cells among (**a**) CD4$^+$CD8$^-$ T cells, (**b**) CD8$^+$CD4$^-$ T cells, (**c**) CD11c$^+$ APCs and (**d**) Ly6G$^+$ neutrophils of WT (open circle) and Hpa-tg (closed circle) mice is shown. The results are shown as means±SEM (n=8 in each group).

Supplementary Table S1: CIA end point day and arthritic score.

Arthritic score of paw swelling		
WT	End day	Arthritic score
B1	85	0
B3	85	0
F2	85	0
F3	85	0
A1	85	2
A2	85	3
A3	85	10
F1	85	23
Hpa-tg	End day	Arthritic score
C2	85	0
D1	85	0
D2	85	0
D3	85	0
E3	85	0
G1	85	0
G2	85	0
C3	85	10
E1	85	14
H2	74*	20
C1	74*	44
H1	64*	46
E2	40*	60

*Mice sacrificed due to the severe symptoms

End point day and arthritic score of paw swelling in CIA WT (light grey) and Hpa-tg (dark grey) mice, with a maximum score of 60. Asterix (*) indicates mice sacrificed early due to severe symptoms.

Supplementary Table S2: Antibodies used for flow cytometer analysis.

Flow cytometer antibodies			
Cell surface markers	Clone	Fluorescent tag	Manufacturer
CD4	RM4-4	FITC	eBioscience
CD25	PC61	PE	eBioscience
CD8	53-6.7	APC-H7	BD
CXCR5	2G8	APC	BD
PD-1	J43	BV605	BD
CD19	1D3	BV605	BioLegend
B220	RA3-6B2	PEcy7	BioLegend
Ly6G	1A8	BV421	BioLegend
CD11c	N418	APC	BioLegend
PDCA-1	927	PE	BioLegend
CD11b	M1/70	FITC	BioLegend
CCR6	29-2L17	PE	BioLegend
Intracellular markers			
Foxp3	FJK-16s	PEcy7	eBioscience
IL-17a	TC11-18H10.1	APC or PE	BioLegend
Helios	22F6	Pacific Blue	BioLegend
Ki-67	16A8	PE	BioLegend

Marker, clone name, fluorescent tag and manufacturer of antibodies used in the study. The table is divided into cell surface (light grey) and intracellular (dark grey) markers.
